# Noradrenergic Hypothesis Linking Neurodegeneration-Based Cognitive Decline and Astroglia

**DOI:** 10.3389/fnmol.2018.00254

**Published:** 2018-07-27

**Authors:** Giampiero Leanza, Rosario Gulino, Robert Zorec

**Affiliations:** ^1^Department of Drug Sciences, University of Catania, Catania, Italy; ^2^Department of Biomedical and Biotechnological Sciences, University of Catania, Catania, Italy; ^3^Laboratory of Neuroendocrinology-Molecular Cell Physiology, Faculty of Medicine, Institute of Pathophysiology, University of Ljubljana, Ljubljana, Slovenia; ^4^Laboratory of Cell Engineering, Celica Biomedical, Ljubljana, Slovenia

**Keywords:** noradrenaline (norepinephrine), cognitive decline, Alzheimer's disease, neurodegeneration, neurogenesis, astroglia

## Abstract

In the past, manipulation of the cholinergic system was seen as the most likely therapeutic for neurodegeneration-based cognitive decline in Alzheimer's disease (AD) (Whitehouse et al., 1982). However, targeting the noradrenergic system also seems a promising strategy, since more recent studies revealed that in post-mortem tissue from patients with AD and other neurodegenerative disorders there is a robust correlation between cognitive decline and loss of neurons from the Locus coeruleus (LC), a system with diffuse noradrenaline (NA) innervation in the central nervous system (CNS). Therefore, the hypothesis has been considered that increasing NA signaling in the CNS will prevent, or at least halt the progression of neurodegeneration and cognitive decline. A hallmark of the age- and neurodegeneration-related cognitive decline is reduced neurogenesis. We here discuss noradrenergic dysfunction in AD-related cognitive decline in humans and its potential involvement in AD pathology and disease progression. We also focus on animal models to allow the validation of the noradrenergic hypothesis of AD, including those based upon the immunotoxin-mediated ablation of LC based on saporin, a protein synthesis interfering agent, which offers selective and graded demise of LC neurons, Finally, we address how astrocytes, an abundant and functionally heterogeneous cell type of neuroglia maintaining homeostasis, may participate in the regulation of neurogenesis, a new strategy for preventing LC neuron loss.

## Introduction

With its widespread efferent projections, the small brainstem nucleus Locus coeruleus (LC) represents the main source of noradrenergic innervation to the entire CNS, and plays a pivotal regulatory role in a variety of physiological processes, including attention, arousal, sleep/wakefulness, consciousness as well as in specific aspects of learning and memory (Amaral and Sinnamon, 1977; Aston-Jones and Cohen, 2005; Benarroch, 2009; Sara, 2009). Notably, increasing clinical and imaging evidences (Peterson and Li, 2018) indicate that LC degeneration constitutes a crucial early event in the pathogenesis of Alzheimer's (AD) and Parkinson's disease and most of the LC-regulated functions have been shown to be severely affected during its progression (Chan-Palay and Asan, 1989; Rub et al., 2001; Wilson et al., 2013; Arendt et al., 2015). Taken together, these observations have pointed at the noradrenergic system as a viable therapeutic target for the treatment of diseases characterized by memory loss and cognitive decline. However, a feasible animal model recapitulating noradrenergic neuronal and terminal fiber loss and its histopathological and cognitive sequelae has not yet been achieved, mainly due to the objective difficulty to selectively and efficiently target LC neurons. This review seeks to briefly summarize noradrenergic dysfunction in AD-related memory loss and its potential involvement in AD pathology and progression. Also, recent findings emerging from our own studies addressing selective immunotoxic ablation of LC neurons and its effects upon cognitive performance, tissue pathology and hippocampal neurogenesis will be briefly outlined. Then we focus into impaired neurogenesis in neurodegeneration and point to the possible contribution of astroglia to this process. Invariably, much relevant literature on the aforementioned topics will not be mentioned here, and we apologize for this.

## Locus coeruleus dysfunction in alzheimer's disease

The existence of an association between noradrenergic depletion and neurodegeneration including AD has long been known (Ishii, 1966; Forno and Alvord, 1971; Mann et al., 1980, 1982; Tomlinson et al., 1981; Iversen et al., 1983), however only in relatively recent years has noradrenergic neuron loss in LC been widely acknowledged as a prominent feature of neurodegeneration and AD, being present often decades prior to the appearance of clinical symptoms, and having been related to neurofibrillary pathology and the severity of cognitive deficits, when overtly present (Haglund et al., 2006; Grudzien et al., 2007; Braak and Del Tredici, 2011a,b; Wilson et al., 2013; Andres-Benito et al., 2017; Peterson and Li, 2018).

There have also been conflicting reports regarding the NA levels in the brain of AD patients. In fact, while some studies have reported marked decline in regional NA brain tissue content, whose magnitude correlated with the severity of cognitive impairments (Martignoni et al., 1992; Nazarali and Reynolds, 1992; Matthews et al., 2002; Chen et al., 2014), others reported no changes (Sparks et al., 1988; Herregodts et al., 1989; Tohgi et al., 1992) or even increased NA levels (Tohgi et al., 1992; Elrod et al., 1997).

Similar conflicting observations in post-mortem brain specimens from AD patients have been reported for adrenergic receptors (Shimohama et al., 1986; Kalaria et al., 1989; Pascual et al., 1992; Leverenz et al., 2001), known to be key mediators of noradrenergic activity, and recently considered relevant candidates as novel therapeutic targets for AD (Chen et al., 2014). These discrepancies, likely reflecting compensatory responses, or their lack, at more advanced stages of the disease (Szot et al., 2006, 2007), have thus driven the need to conclusively dissect the exact role played by the noradrenergic system in the cognitive sequelae and pathogenesis that characterize AD and neurodegeneration in general. During the last decades, numerous animal studies and reviews (Mather and Harley, 2016; Borodovitsyna et al., 2017; Gannon and Wang, 2018) have provided valuable insights into the factors underlying the disease. It has been shown, for example, that - possibly via their direct connections to the prefrontal cortex and hippocampus - LC neurons have a fundamental role in sustaining behavioral responsiveness upon exposure to relevant, reward-predicting, stimuli (Bouret and Sara, 2004; Hagena et al., 2016), including those related to working memory (Milstein et al., 2007; Coradazzi et al., 2016). Furthermore, LC neuron degeneration offers a major contribution to AD pathogenesis and progression (Braak et al., 2011; Iba et al., 2015). In fact, a prevailing hypothesis for noradrenergic neuron and fiber depletion in AD holds that LC neurons are uniquely susceptible to tau toxicity (Chandler et al., 2014) and are especially vulnerable to oxidative stress, possibly owing to their high bioenergetic needs (Sanchez-Padilla et al., 2014). In such scenario, accumulation of abnormally phosphorylated tau in LC neurons and its spreading to most of the brain due to the extremely diffuse efferent projections, would account for the progression of the disease (Braak and Del Tredici, 2015), and the resulting neuronal degeneration and cognitive impairments.

## Experimental animal paradigms to study noradrenergic dysfunction

**Figure 1:**
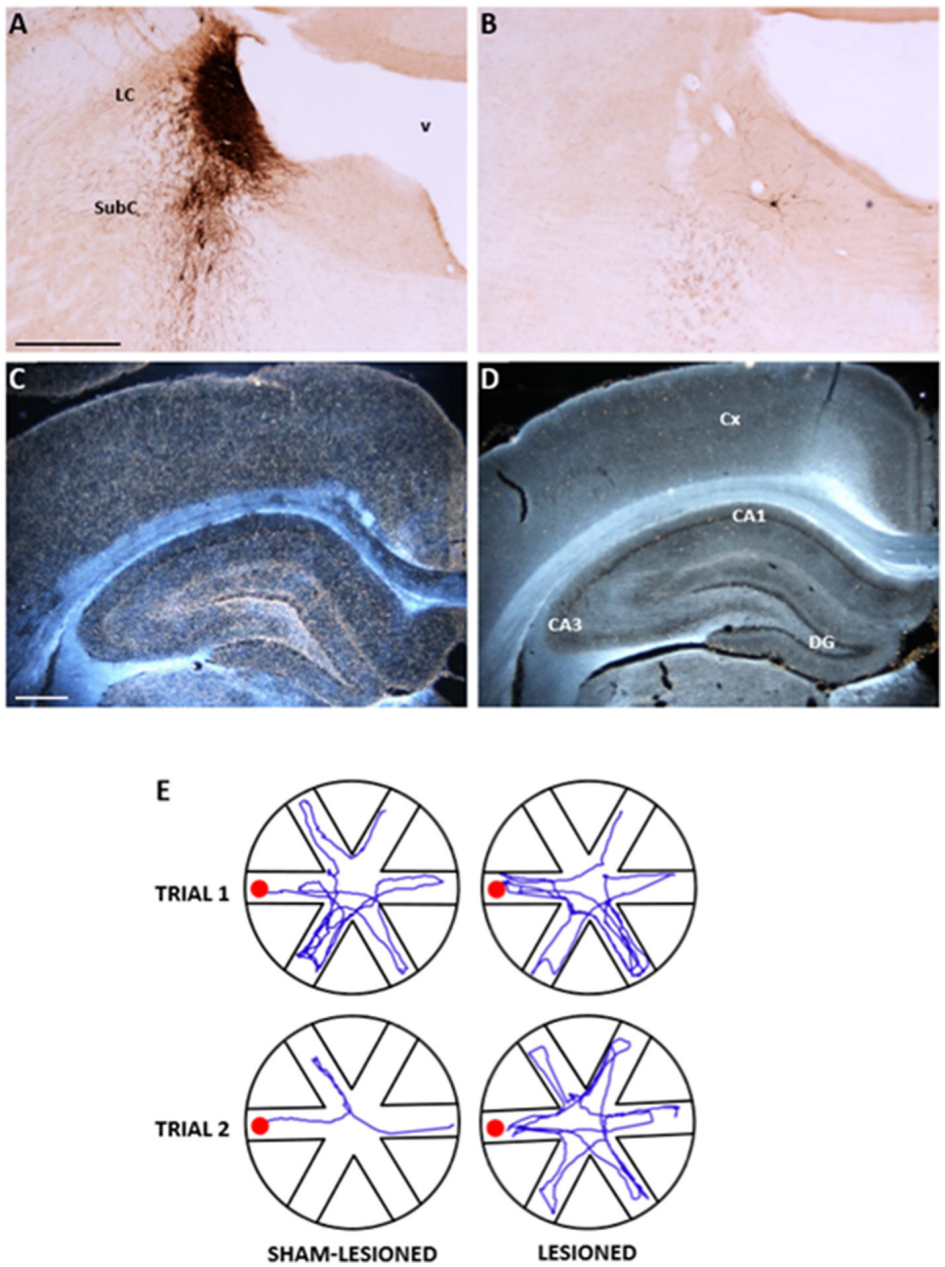
Anatomical and functional effects of the selective noradrenergic lesion in young adult Sprague-Dawley rats. **(A–D)**: Photomicrographs showing the effects of the anti-DBH immunotoxin, injected bilaterally into the LC, on noradrenergic neurons in the LC/SubC **(A,B)** and on the DBH-immunoreactive terminal innervation in the parietal cortex and hippocampus (**C,D**, in dark field). Note in **(B,D)** the nearly complete loss of immunoreactive neurons and fibers induced by the lesion, compared to the normal patterns in the specimens from a sham-lesioned animal **(A,C)**. In **(E)**, the actual swim paths taken by representative sham-lesioned and lesioned animals undergoing the Radial Arm Water Maze (RAWM) task for working memory are illustrated. The sham-lesioned animal rapidly learns the task and significantly improves its performance from the first to the second trial, whereas the lesioned animal does not. LC, locus coeruleus; SubC, subcoeruleus, v fourth ventricle, CA, cornu ammonis of the hippocampus; DG, dentate gyrus. Scale bars in **(A,C)**: 500 μm. Adapted from Coradazzi et al. (2016).

Animal studies have so far been extremely helpful to dissect the importance of NA in cognition and in the pathological events associated to its loss. Experimental manipulation of the noradrenergic system by e.g., pharmacological blockade (Mair et al., 2005; Khakpour-Taleghani et al., 2009), lesioning with N-(2-chloro-ethyl)-N-ethyl-2- bromobenzylamine (DSP4), reportedly an LC-selective neurotoxin (Lapiz et al., 2001; Sontag et al., 2008) or the knockout of the dopamine-β-hydroxylase (DBH) gene (Thomas and Palmiter, 1997; Marino et al., 2005; Hammerschmidt et al., 2013) have all resulted in impaired performance in several learning and memory tasks, demonstrating the existence of an association between NA loss and disturbances in various aspects of cognition. However, in many cases, the impairments observed in these studies have appeared rather inconsistent, both in efficiency and selectivity, thus highlighting the potential limitations inherent to each method (Sontag et al., 2008; Khakpour-Taleghani et al., 2009; Szot et al., 2010; Gannon et al., 2015). In fact, pharmacological agents lack anatomical and neurochemical resolution, acting on most monoaminergic neurons and cells throughout the central and peripheral nervous system. Likewise, DSP4 does not seem to be specific for noradrenergic neurons and has been shown to produce only a modest noradrenergic neuron loss, at best. Finally, although DBH (–/–) knockout mice have provided the unique opportunity to precisely assess the effects of NA loss *per se*, with respect to the various modulators produced and released by the very same LC neurons, they do not seem to offer the possibility to obtain partial or graded neurotransmitter depletions. In light of these limitations, we have chosen an alternative lesioning approach based on the use of the immunotoxin anti-DBH-saporin (Picklo et al., 1994), able to target noradrenergic neurons in the LC with unprecedented selectivity and efficiency. This immunotoxin results from the conjugation of saporin, a powerful ribosome-inactivating plant lectin extracted from *Saponaria officinalis* (Caryophyllaceae) (Lappi et al., 1985; Barthelemy et al., 1993) to a monoclonal antibody raised against DBH (the enzyme converting dopamine to NA) that, in addition to its main localization in the cytosol, is also expressed at the plasma membrane surface of noradrenergic neurons (Weinshilboum, 1978; Studelska and Brimijoin, 1989). Due to its structure, saporin cannot enter the cell (Contestabile and Stirpe, 1993), but when coupled to a carrier molecule (e.g., an antibody), is able to specifically bind a surface antigen protein (such as DBH, in this case), the toxin gains access to the cytosol and binds to the ribosomal 60S subunit, interfering with protein synthesis, and soon leading to cell death (Wiley and Kline, 2000). In initial anatomical investigations, the immunotoxin, infused into the lateral ventricles of either adults (Wrenn et al., 1996) or developing rats (Coradazzi et al., 2010) has been observed to produce highly specific and dose-dependent depletions of LC neurons, with no effects on other cholinergic, dopaminergic or serotonergic neuronal populations (Figure 1). Notably, the possibility to induce a partial or total noradrenergic loss (by varying the injected dose) makes this immunotoxic approach extremely suitable to address compensatory events within the noradrenergic projection system, in addition to providing an excellent tissue environment for the survival and integration of implanted NA-rich progenitors (Coradazzi et al., 2010).

## Noradrenaline and adult hippocampal neurogenesis

The subgranular zone of the hippocampal dentate gyrus is one of the brain regions where generation of neural progenitor cells occurs thoughout life in various species, including humans (Altman and Das, 1965; Cameron et al., 1993; Eriksson et al., 1998; Gould et al., 1998). From here, proliferating newborn cells migrate to the granule cell layer, where they differentiate into neurons and glia and functionally integrate into the local tissue environment (Markakis and Gage, 1999; Carlen et al., 2002; van Praag et al., 2002). Many factors have been observed to affect hippocampal neurogenesis, including environmental or stressful stimuli (Dranovsky and Hen, 2006), various kinds of hormones and drugs (Duman et al., 2001), as well as neurotransmitter activity (Brezun and Daszuta, 1999; Mohapel et al., 2005, 2006; Aztiria et al., 2007; Walker et al., 2008). Notably, the hippocampus is the region where explicit memories are apparently acquired and consolidated (Murchison et al., 2004), and one of the brain areas receiving the densest LC-derived noradrenergic innervation (Swanson and Hartman, 1975). Moreover, hippocampal neurogenesis has been proposed to underlie some of the behavioral effects of antidepressant drugs (Santarelli et al., 2003; Warner-Schmidt and Duman, 2006; Airan et al., 2007), whose actions are mainly exerted by increasing extracellular levels of serotonin and/or noradrenaline (Fuller et al., 1994; Sacchetti et al., 1999). It is therefore not surprising that several studies have begun to investigate the contribution of LC neurons to the regulation of hippocampal neurogenesis, and have generally reported a permissive role for NA upon hippocampal neurogenesis (Kulkarni et al., 2002; Jhaveri et al., 2010, 2014; Masuda et al., 2012). Again, however, none of the lesion or pharmacological manipulations adopted in these investigations proved to be region-or transmitter specific, nor were any of the observed effects analyzed also in terms of impact upon cognitive performance. In our study using selective and discrete immunolesioning of LC neurons, associated with a series of hippocampus-dependent spatial navigation tasks (Coradazzi et al., 2016), we found severe deficits in working memory, which correlated with the magnitude of hippocampal noradrenergic depletion and the lesion-induced reduction in the numbers of proliferating cells within the dentate gyrus. Notably, no changes were detected in reference memory abilities, nor did the lesion affect long-term survival or differentiation of granule cell progenitors (Coradazzi et al., 2016). Thus, the noradrenergic regulation of complex aspects of cognitive function (e.g., those related to working memory) may take place via the proliferation of progenitor cells in the hippocampal dentate gyrus.

## Noradrenaline, astroglia and neurogenesis

**Figure 2:**
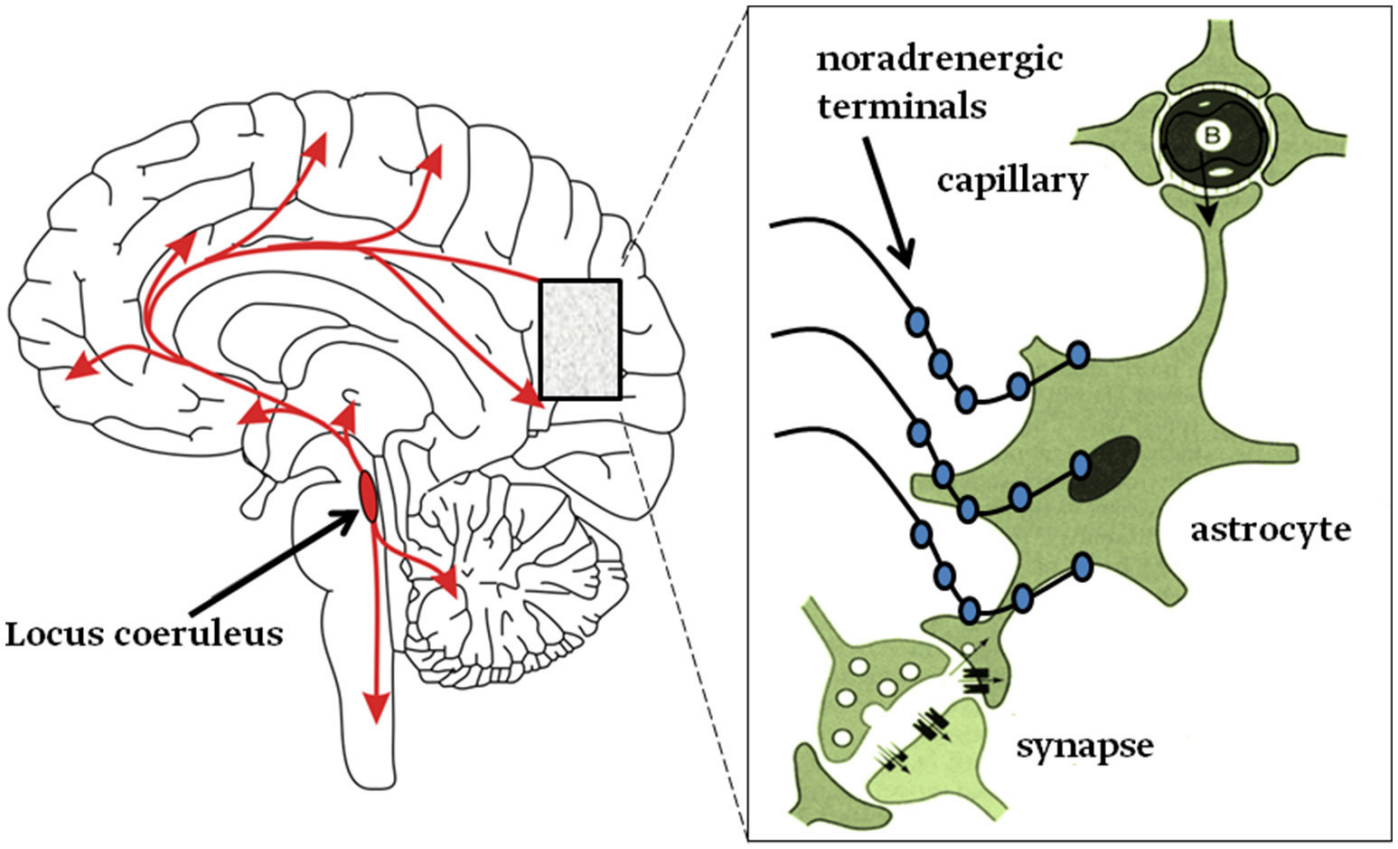
Graphical abstract. **Left**: neurons from the Locus coeruleus (LC) project axons to most, if not all, areas of the brain and into the spinal cord as denoted by the arrows. **Right**: inset magnifies the LC nerve endings with varicosities (swellings on noradrenergic nerve terminals) from which NA is released and mainly activates astroglia (Aoki, 1992; Sherpa et al., 2016). About half of these terminals do not form tight contacts with target cells, resembling synapses (Feinstein et al., 2016).

It appears that noradrenergic receptors are densely present in astroglia (Aoki, 1992), therefore it is likely that NA may affect neuronal circuits via astroglia (Ding et al., 2013; Paukert et al., 2014; Pankratov and Lalo, 2015; Gao et al., 2016; Dong et al., 2017) (Figure 2. graphical abstract). Current view holds that neurodegeneration in AD is a consequence of neuron-specific deficits. However, it is more likely that preceding or concomitant changes in neuroglia may also contribute to this process (Heneka et al., 2015; Verkhratsky and Parpura, 2015; De Strooper and Karran, 2016; Rodriguez-Vieitez et al., 2016; Stenovec et al., 2016; Verkhratsky et al., 2016). Astroglia, a type of neuroglia, consisting also of oligodendroglia, microglia and NG2 cells, are functionally and morphologically heterogeneous, and are involved in sustaining brain homeostasis at cellular and whole organ levels, by regulating extracellular levels of ions and neurotransmitters, by controlling vascular and metabolic functions, the integrity of blood-brain barrier (BBB) (Terry, 2000; Giaume et al., 2007; Kano and Hashimoto, 2009; Heneka et al., 2010; Nedergaard et al., 2010; Parpura and Zorec, 2010; Verkhratsky and Nedergaard, 2014, 2018; Zorec et al., 2018). Importantly, astrocytes are essential in orchestrating defense in the CNS as well; pathological states in the CNS lead to reactive astrogliosis, a process that contains and isolates events taking place in the damaged brain regions. Moreover, reactive astrogliosis is also augmenting post-damage regeneration and repair of brain tissue (Parpura et al., 2012; Pekny et al., 2016; Verkhratsky et al., 2016); however, under certain conditions reactive astrogliosis can be neurotoxic (Liddelow et al., 2017). Astrocytes were also termed gliocrine cells (Vardjan and Zorec, 2015), since they secrete gliosignalling molecules into the extracellular space and are then convectively distributed thoroughout the brain by the glymphatic system (at least in mice), responsible for waste removal (Thrane et al., 2014). Changes in these complex functions of astroglia may lead to a homeostatic failure, leading to disease (Verkhratsky and Parpura, 2015). Hence, primary defect in homeostatic astroglia may lead to a secondary defect in neurons. Interestingly, the role of neuroglial cells in dementia and AD was noted already at least a century ago by A. Alzheimer, who observed glial cells in the proximity of damaged neurons (Alzheimer, 1907, 1910; Strassnig and Ganguli, 2005).

In tissue from post-mortem patients with AD, several morphological observations were made, including astroglial hypertrophy, associated with reactive astrogliosis, the hallmark of which is increased expression of cytoskeletal glial fibrillary acidic protein (GFAP) and S100, a Ca^2+^-binding protein; these changes in astrocytes were seen associated with senile plaques (Beach and McGeer, 1988; Griffin et al., 1989; Nagele et al., 2004; Mrak and Griffin, 2005; Verkhratsky et al., 2014). Further, studying patients with AD by imaging methods revealed distinct time- and brain-specific morphological alterations (Rodriguez-Vieitez et al., 2016). Similar changes occurred in an animal mouse model of familial AD as well, including astroglial atrophy in addition to hypertrophy in certain areas of the CNS (Olabarria et al., 2010, 2011; Yeh et al., 2011; Kulijewicz-Nawrot et al., 2012). The appearance of asthenic astrocytes appeared to preced the presence of senile plaques; asthenic astrocytes were at first observed in the entorhinal cortex, an area affected early in AD pathology (Yeh et al., 2011). Ideally, studies of pathological developments associated with neurodegeneration and AD would be best in humans, but these experiments are challenging. Recent attempts to classify protein astrogliopathies, including deposition of amyloid-β, prion protein, tau, α-synuclein, and transactive response DNA-binding protein 43 (TDP-43), demonstrated that these are present in human neurodegenerative diseases (Kovacs et al., 2017). However, animal models seem still very valuable, particularly where many aspects of this pathology can be, to some extent, reproduced, and the time emergence of AD-like characteristic take a much shorter time to develop, providing certain opportunities for studying AD-related neuropathology experimentally. Not only mice, there are several other animal models of AD including nematodes, fruit flies, rabbits, canines, and non-human primates; in each of the models different aspects of AD properties are manifested (reviewed in Woodruff-Pak, 2008). The selective ablation of LC in rats, as presented in previous chapters, offers interesting insights to be explored as a novel animal model of AD and neurodegeneration, not only for functional cognitive impairments (Coradazzi et al., 2016), but also for morphological alterations and aspects of neurogenesis related to astroglia.

Neuron degeneration in the LC during early stages of AD (Heneka et al., 2006) may lead to reduced levels of NA, which is known to be a generic inhibitor of neuroinflammation (De Keyser et al., 2004) and remodeling of the neurovascular unit (del Zoppo, 2009). Therefore, reduced noradrenergic innervation in the CNS may affect the progression of AD through reduced inhibition of neuroinflammation, a tissue remodeling process. This likely operates via a reduced NA contribution of astrocyte adrenergic excitation. Although NA released by the LC neurons acts through α- and β-adrenergic receptors (α/β-ARs), which are expressed in neurons, microglia and astrocytes, but it is the latter cell type that exhibits a high density of ARs, especially the β-ARs (Aoki, 1992) and thus represents a key cell type mediating adrenergic effects on brain tissue. Indeed, activation of α-ARs stimulates Ca^2+^ signaling in astrocytes (Salm and McCarthy, 1989; Kirischuk et al., 1996; Horvat et al., 2016); experiments *in vivo* reported Ca^2+^ waves propagating through astroglial syncytia after stimulating the LC in anesthetized animals (Bekar et al., 2008). In awake animals, electrical stimulation of LC triggered (via activation of α_?_-ARs) widespread and synchronous astroglial Ca^2+^ signals in practically all astrocytes in the field of study (Ding et al., 2013). This phenomenon may be taken as an event resetting neural networks (Bouret and Sara, 2005). In AD, astrocytic Ca^2+^ signaling is impaired (Lim et al., 2014; Stenovec et al., 2016), which may affect the clearance of Aβ deposits (Mattson, 2004) and subsequent glutamate toxicity (Mattson and Chan, 2003; Mattson, 2004).

Whenever NA is released, not only α-ARs, but also β-ARs are stimulated, although the effects appear in different time-domains (Horvat et al., 2016). These latter receptors stimulate second messenger cAMP, which affects glycogenolysis (Prebil et al., 2011; Kreft et al., 2012), a likely source of glutamate for memory consolidation (Gibbs et al., 2010). Glycogen, an energy reserve, present in astrocytes, but not in neurons (Barros, 2013; Oe et al., 2016), is likely consumed for processes related to morphological plasticity controlled by β-AR/cAMP signaling in astroglia (Vardjan et al., 2014). Not only in memory formation (Zorec et al., 2015), astroglial β-AR/cAMP-dependent morphological changes are central for astrocytic cell oedema attenuation (Vardjan et al., 2016).

Activation of ARs on astrocytes may also affect neurogenesis through neuronal metabolic support by astroglia. Reduced support due to lower NA levels may in AD result in reduced neurogenesis. Astrocytes are the site of aerobic glycolysis, a special metabolic adaptation, present in tissues exhibiting cell division and morphological plasticity. This non-oxidative utilization of glucose, takes place even in the presence of adequate levels of oxygen; it is also known as “the Warburg effect” (Vander Heiden et al., 2009). It is also a characteristic of cancer cells (Salcedo-Sora et al., 2014), hence aerobic glycolysis seems a universal property needed for tissue enlargement and cell shape remodeling.

Astroglial aerobic glycolysis, with the end product L-lactate, is regulated by NA. During exercise, sensory stimulation, alertness and in some pathophysiological states astroglial L-lactate production is up-regulated, requiering the activation of LC neurons (Dienel and Cruz, 2016; Feinstein et al., 2016). LC neurons respond to L-lactate, generated by astroglia when NA activates ARs on astroglial plasma membrane, with elevated electrical activity, an interesting form of communication between the somata of the two cell-types (Tang et al., 2014). It is possible that astroglial L-lactate may also stimulate LC axons away from the somata; as it was recently demonstarted that transcranial direct current stimulation induces NA-dependent elevation of second messenger Ca^2+^ in astrocytes (Monai et al., 2016). Therefore, degenerated LC neurons in AD fail to adequately stimulate astroglia, a source of necessary support for the metabolic needs of neuronal networks, contributing to attenuated neurogenesis. Hypometabolic manifestation is observed clinically in patients with AD (Rodriguez-Vieitez et al., 2016).

Cholesterol is an important building block of membranes and astroglial aerobic glycolysis is likely linked to cholesterol homeostasis in the brain, the most cholesterol-rich organ in the body (Lütjohann et al., 1996). There is little exchange of cholesterol molecules by circulating lipoproteins, between the brain and systemic circulation, since lipoproteins are unable to pass the blood–brain barrier (BBB). Interestingly, the brain exhibits its own cholesterol synthesis, which occurs mainly in glia (Mauch et al., 2001)). Brain cholesterol is not only synthesized *de novo* in the brain, it is also modified/degraded by enzymatic conversion to 24(*S*)-hydroxycholesterol (Lütjohann et al., 1996), a form that can readily cross the BBB, a major route of cholesterol exit from the brain.

Cholesterol metabolism is linked to the synthesis of the neurosteroids including allopregnanolone, a potent stimulator of neural progenitor cell survival reversing the progress of disease in the 3xTgAD mouse model (Singh et al., 2012). Thus, it is possible to speculate that the hypometabolic state in AD, due to impairment of LC-mediated adrenergic stimulation of astroglia, results in a reduced provision of metabolic intermediates and in attenuated neurosteroid-mediated maintenance of neurogenesis, an avenue that will have to be confirmed experimentally in the future.

## Future prospects and therapy options

Based on the above observations of the relationship between central noradrenergic depletion and a series of AD-related changes, including cognitive disturbances, and tissue pathology, it is not surprising that much interest has recently mounted concerning the possibility to restore extracellular NA levels in the brain as a prerequisite to ameliorate cognitive performance as well as to promote cell protection and neurogenesis. Several potential therapeutic venues have been explored so far: one holds that voluntary physical exercise may improve memory by enhancing NA release from LC neurons (Segal et al., 2012), an effect possibly mediated by β-ARs (Van Hoomissen et al., 2004; Ebrahimi et al., 2010). Another study has addressed the possible restorative effects of atomoxetine (a NA reuptake inhibitor) in AD patients undergoing anticholinesterase therapy, reporting no clear-cut cognitive improvements (Mohs et al., 2009), possibly due to a poor effectiveness of the NA uptake inhibitor on LC which is already severely depleted of its neurons as a result of the disease (Braun et al., 2014; Braun and Feinstein, 2017). A similar lack of cognitive improvements has been reported in aged healthy patients following treatment with guanfacine, an α_2_-AR agonist (Van Dyck, 2014; http://ClinicalTrials.gov) that, if anything, should reduce, rather than increase NA levels (Starke, 2001). Indeed, α_2_-AR antagonists appear to prevent age-related spatial working memory impairments in a transgenic AD mouse model (Scullion et al., 2011). In spite of these inconsistencies, the possibility to ameliorate cognitive performance and cell protection by enhancing noradrenergic neurotransmission remains an exciting prospect: in a recent investigation (Pintus et al., 2018) we have implanted embryonic noradrenergic progenitors bilaterally into the hippocampus of rats whose LC neurons had been almost completely and selectively depleted. We reasoned that if the loss of noradrenergic innervation to the hippocampus was necessary to induce measurable cognitive impairments, its restoration promoted by the implanted embryonic LC neurons should be sufficient to ameliorate/reverse them. As expected (and consistent with our previous findings Coradazzi et al., 2016), the LC lesion induced severe deficits in working memory which were seen fully reversed (up to normal) in transplanted animals and significantly reinstated by a second lesion ablating the implanted neuroblasts. Interestingly, the transplant-promoted noradrenergic reinnervation also normalized the nuclear expression of the transactive response DNA-binding protein 43 (TDP-43) in various hippocampal subregions, whose cytoplasmic (i.e., pathological) occurrence appeared dramatically increased as a result of the lesions. These findings therefore provide support to the view that cognitive and histopathological changes observed in AD patients may require concurrent loss of ascending regulatory noradrenergic inputs from LC and that NA replenishment may be an effective intervention to slow down and/or reverse cognitive decline and tissue pathology, including neurogenesis. Moreover, the NA replenishment may operate via astroglial adrenergic mechanism that include metabolic and neurotrophic support of neural networks.

## Concluding remarks

Noradrenergic neurons in the LC and their widespread efferent connections play a central role in normal cognition and their disruption is increasingly believed to be critically associated with severe memory loss and neurodegeneration in general (Wilson et al., 2013; Feinstein et al., 2016; Bharani et al., 2017; Satoh and Iijima, 2017). Notably, the presence of functional and densely present adrenergic receptors on astrocytes (Aoki, 1992), whose activation triggers metabolic and energetic responses supporting synaptic functioning and plasticity (Zorec et al., 2017), strongly suggests that many, if not all, the NA-mediated processes may take place also via an action on astrocyes. Thus, addressing astrocytes as viable targets for neurological diseases related to noradrenergic dysfunction is an interesting issue with fundamental therapeutic implications, warranting further research.

## Author contributions

GL, RG, and RZ conceived the concept of paper, wrote and edited the manuscript.

### Conflict of interest statement

The authors declare that the research was conducted in the absence of any commercial or financial relationships that could be construed as a potential conflict of interest.
